# Relationship between the Hogarth “line of beauty” and the female gluteal profile in western idealized aesthetics

**DOI:** 10.1016/j.jpra.2026.06.004

**Published:** 2026-06-18

**Authors:** Edoardo Raposio, Elisa Bertulla

**Affiliations:** a Plastic Surgery Chair, Department of Surgical Sciences and Integrated Diagnostics (DISC), University of Genova, Italy; b Plastic and Reconstructive Surgery Division, IRCCS Azienda Ospedaliera Metropolitana, Genova, Italy

The increasing popularity of cosmetic procedures aimed at modifying the female body contour—particularly gluteal augmentation—has renewed interest in identifying principles, reference lines, and geometric constructs that may help describe, as objectively as possible, aesthetic ideals used in planning and outcome assessment.^1^, ^2^, ^3^, ^4^, ^5^

The “line of beauty” is an art-theoretical concept referring to an S-shaped (“serpentine”) curve that may appear within an object, outline its boundary, or emerge as an implied contour created by compositional elements. The concept is generally attributed to William Hogarth and constitutes a central element of his aesthetic theory presented in *The Analysis of Beauty* (1753). In Hogarth’s account, serpentine curves convey liveliness and visual interest when contrasted with straight, parallel, or right-angled lines.

Although the serpentine line is not necessarily intended to govern an entire composition, it is frequently discussed in relation to localized subject matter, including the human figure. Within Hogarth’s framework, one particular S-shaped configuration (Fig. 1) has been proposed as especially representative of aesthetically pleasing forms. Lumbar curvature approximating a biomechanical optimum for pregnancy-related demands is associated with higher attractiveness ratings in female profiles, a finding that may conceptually align with Hogarth’s emphasis on the S-shaped contour.

**Fig. 1:**
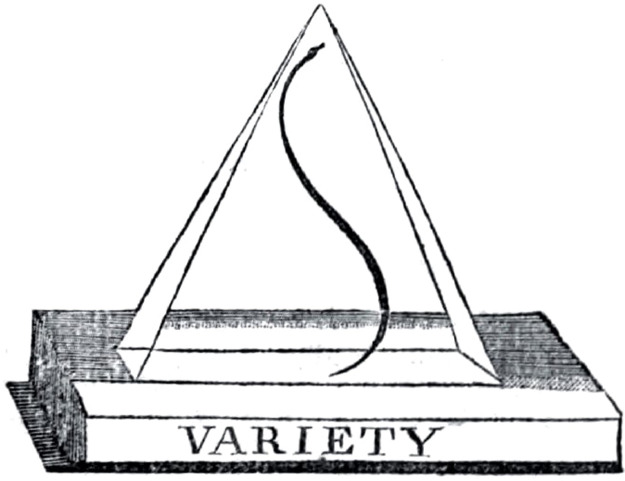
William Hogarth, illustration of the line of beauty.

In this study, we investigated whether a Hogarth-like S-shaped contour can be observed in classical Western paintings depicting idealized female figures in profile. We screened 2880 paintings; 16 met the inclusion criteria (young female figures depicted in profile and presented as idealized within the artwork). In all included images, the body outline closely approximated the superimposed Hogarth curve. A representative example is provided in Fig. 2. We suggest that this observation may contribute, as one element within a broader framework, to ongoing efforts to formalize geometric descriptors of idealized female body aesthetics.

**Fig. 2:**
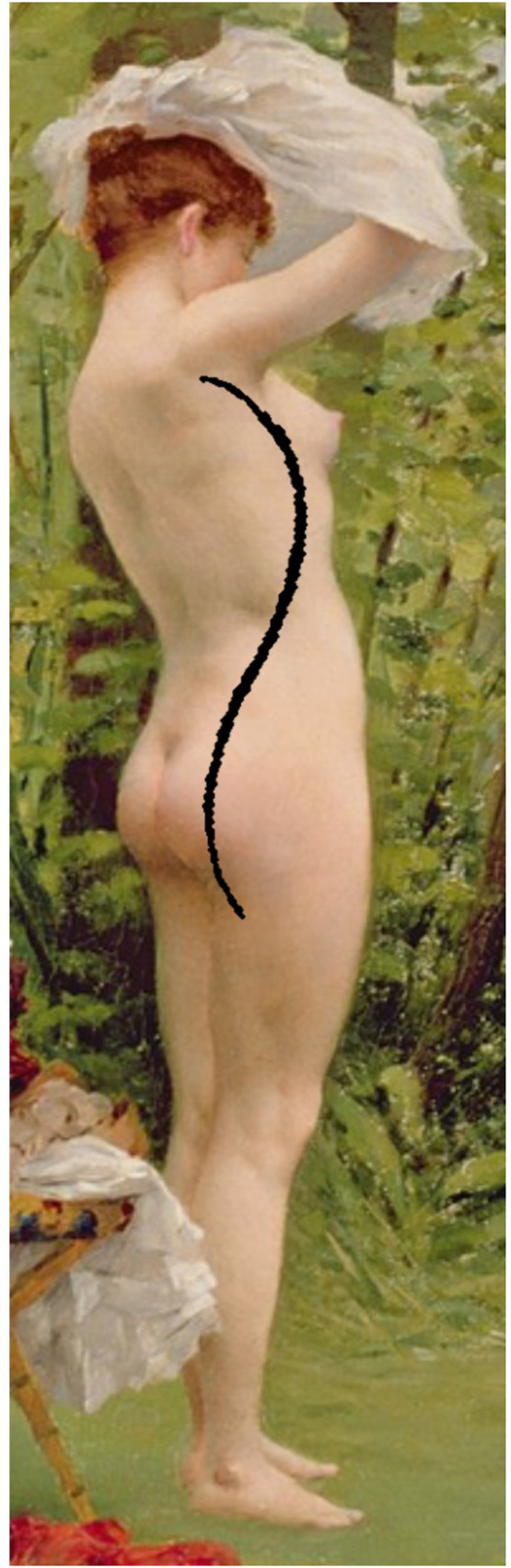
Jules Scalbert, *The Bathers*, approx. 1910, Oil on canvas, Josef Mensing Gallery, Hamm-Rhynern, Germany, with the line S of Hogarth superimposed.

An important limitation of the present work is that we exclusively examined paintings produced by European artists and depicting European women. Given that standards of female beauty were and continue to be, at least in part, culture-specific and variable across populations and societies, the considerations presented here primarily concern European culture and its corresponding ideals of female beauty.

## Ethical approval

Not required.

## Clinical Trial number

Not applicable.

All the figures are in the Public Domain.

## Funding

None.

## Declaration of competing interest

None.
